# The genome of *Romanomermis culicivorax*: revealing fundamental changes in the core developmental genetic toolkit in Nematoda

**DOI:** 10.1186/1471-2164-14-923

**Published:** 2013-12-27

**Authors:** Philipp H Schiffer, Michael Kroiher, Christopher Kraus, Georgios D Koutsovoulos, Sujai Kumar, Julia I R Camps, Ndifon A Nsah, Dominik Stappert, Krystalynne Morris, Peter Heger, Janine Altmüller, Peter Frommolt, Peter Nürnberg, W Kelley Thomas, Mark L Blaxter, Einhard Schierenberg

**Affiliations:** 1 Zoologisches Institut, Universität zu Köln, Cologne, NRW, Germany; 2 Institute of Evolutionary Biology, School of Biological Sciences, The University of Edinburgh, Edinburgh, Scotland, UK; 3 Institute für Entwicklungsbiologie, Universität zu Köln, Cologne, NRW, Germany; 4 Hubbard Center for Genome Studies, University of New Hampshire, Durham, NH, USA; 5 Cologne Center for Genomics, Universität zu Köln, Cologne, NRW, Germany

**Keywords:** Nematode, Genome, Evolution, Development, Caenorhabditis, Mermithida, Romanomermis

## Abstract

**Background:**

The genetics of development in the nematode *Caenorhabditis elegans* has been described in exquisite detail. The phylum Nematoda has two classes: Chromadorea (which includes *C. elegans*) and the Enoplea. While the development of many chromadorean species resembles closely that of *C. elegans*, enoplean nematodes show markedly different patterns of early cell division and cell fate assignment. Embryogenesis of the enoplean *Romanomermis culicivorax* has been studied in detail, but the genetic circuitry underpinning development in this species has not been explored.

**Results:**

We generated a draft genome for *R. culicivorax* and compared its gene content with that of *C. elegans*, a second enoplean, the vertebrate parasite *Trichinella spiralis*, and a representative arthropod, *Tribolium castaneum*. This comparison revealed that *R. culicivorax* has retained components of the conserved ecdysozoan developmental gene toolkit lost in *C. elegans*. *T. spiralis* has independently lost even more of this toolkit than has *C. elegans*. However, the *C. elegans* toolkit is not simply depauperate, as many novel genes essential for embryogenesis in *C. elegans* are not found in, or have only extremely divergent homologues in *R. culicivorax* and *T. spiralis*. Our data imply fundamental differences in the genetic programmes not only for early cell specification but also others such as vulva formation and sex determination.

**Conclusions:**

Despite the apparent morphological conservatism, major differences in the molecular logic of development have evolved within the phylum Nematoda. *R. culicivorax* serves as a tractable system to contrast *C. elegans* and understand how divergent genomic and thus regulatory backgrounds nevertheless generate a conserved phenotype. The *R. culicivorax* draft genome will promote use of this species as a research model.

## Background

Nematodes have a generally conserved body plan. Their typical form is dictated by the presence of a single-chamber hydroskeleton, where longitudinal muscles act against an inextensible extracellular cuticle. The conservation of organ systems between nematode species is even more striking, with, for example, the nervous system, the somatic gonad and the vulva having very similar general organisations and cellular morphologies across the phylum. It might be thought that these similarities arise from highly stereotypical developmental programmes, but comparative studies challenge this “all nematodes are equal” view.

**Figure 1:**
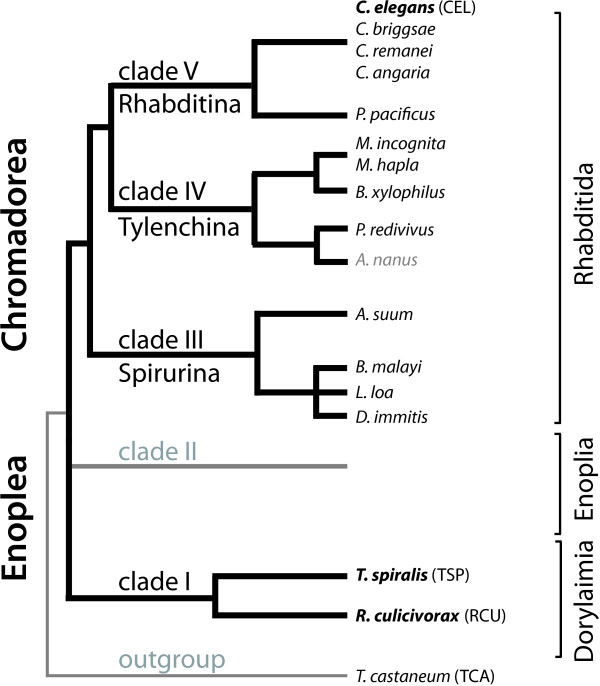
**A simplified phylogenetic tree of the phylum Nematoda.** The phylogeny, simplified from [3,4], emphasises the position of the main study species *R. culicivorax*, *T. spiralis* and *C. elegans*. Species with a published genome and mentioned in Table 1 are in black. Currently no genomic data are available for Enoplia (Clade II). The order of branching of the basal nodes of Nematoda is currently unresolved.

Embryonic development of the nematode *Caenorhabditis elegans* has become a paradigmatic model for studying developmental processes in animals, including early soma-germline separation, fate specification including inductive interactions, and tissue-specific differentiation. The particular mode of development of *C. elegans* is distinct within the major metazoan model organisms, but much of the regulatory logic of its development is comparable to that observed in other phyla. One key aspect in which *C. elegans* differs from vertebrate and arthropod models is that *C. elegans* shows a strictly determined development [1], with a largely invariant cell-lineage giving rise to predictable sets of differentiated cells [2]. Inductive cell-cell interactions are, nevertheless, essential for its correct development [1]. *C. elegans* is a rhabditid nematode, one of approximately 23,000 described and 1 million estimated nematode species. Molecular and morphological systematics of the phylum Nematoda identify two classes: Chromadorea (including Rhabditida, and thus *C. elegans*), and Enoplea (subdivided into Dorylaimia and Enoplia) [3,4] (Figure 1). *C. elegans* is a chromadorean, and most investigation of developmental biology of nematodes has been carried out on Chromadorean species. The first description of the early embryonic cell-lineage of a nematode, that of *Ascaris* (Spirurina within Chromadorea) in the 1880’s [5,6], conforms to the *C. elegans* model. Early development across all suborders of the Rhabditida is very similar [7,8]. In general, only minor variations of the division pattern observed in *C. elegans* have been described in these nematodes [9,10], including heterochrony in the timing of cell divisions, and restrictions in cell-cell interaction due to different placement of blastomeres in the developing embryo. From these observations it might be assumed that all nematodes follow a *C. elegans*-like pattern of development. However, deviations from the *C. elegans* pattern observed in other rhabditid nematodes show that the strictly determined mode of development is subject to evolutionary change, making it particularly attractive for the study of underpinning regulatory logic of developmental mechanisms. Indeed, a greater role for regulative interactions in early development has been demonstrated in another rhabditid, *Acrobeloides nanus* (Tylenchina) [11,12].

**Table 1:** Genome statistics

**Species**	**Approximate **^***#***^	**Estimated**	**Median **^***‡***^	**Median **^***‡***^	**GC**	**Source**
	**genome size**	**repeat content**	**exon length**	**intron length**	**content**	
*C.elegans*	100Mb	17% (16.5%)	145bp	69bp	38%	[17,18]
*P. pacificus*	165Mb	15.3% (17%)	85bp	141bp	42%	[20,25]
*A. suum*	334Mb	4.4%	144bp	907bp	37.9%	[21,40]
*B. malayi*	95Mb	16.5% (15%)	140bp	219bp	30%	[22]
*B. xylophilus*	69Mb	22,5%	183bp	69bp	40%	[25]
*M. incognita*	∼200Mb	36,7%	136bp	82bp	31%	[24]
*T. spiralis*	63Mb	19.8% (18%)	128bp	283bp	34%	[27]
*R. culicivorax*	>270Mb	48.2%	161bp	405bp	36%	this work

Repeat content of different nematode genomes appears not to be directly correlated with genome size. Re-calculation in selected genomes shows little deviance from published data (in parentheses) ^∗^and thus indicates the validity of our inference for *R. culicivorax*.

^∗^For *B. xylophilus* and *M. incognita* only reference data is given as the same programs were used for initial inference (see references); *A. suum* not re-calculated.

^*#*^*M. incognita* genome size given as 86Mbp in [24] has been re-estimated to about 150Mbp (E. Danchin pers. comm.).

^*‡*^Median lengths for *A. suum* and *T. spiralis* were calculated in this work as these data are not given in the cited publications.

Regulative development is common among Metazoa, and is also observed in other Ecdysozoa, including Arthropoda. Indeed, in several enoplean species, early embryos have been found to not display polarised early divisions, arguing against a strongly determined mode of development in this group [13,14]. The determined mode found in *C. elegans* is thus likely to be derived even within Nematoda [15], implying that the core developmental system in Nematoda has changed, while maintaining a very similar organismal output. This phenomenon, termed “developmental system drift” [16], reveals independent selection on the developmental mechanism and the final form produced.

To explore the genetics of development of enoplean and other non-rhabditid nematodes requires tractable experimental systems with a suitable set of methodological tools and extensive genomic data. While *C. elegans* and its embryos are relatively easily manipulated and observed, and the *C. elegans* genome has been fully sequenced [17], embryos from the Enoplia and Dorylaimia are much harder to culture and manipulate. Few viable laboratory cultures exist and obtaining large numbers of embryos from wild material is difficult. Functional molecular analysis of most nematodes, in particular Enoplea, is further hindered by the lack of genetic tools such as mutant analysis or gene-knockdown via RNAi. Performing detailed comparative experimental embryology on a phylogenetically representative set of species across the phylum Nematoda thus remains a distant goal.

The genetic toolkit utilised by a species is represented in its genome, and direct assessment of the genetic capabilities of an organism can thus be assessed through analysis of genome data. Using the background knowledge of pathways and modules used in other taxa, the underpinning logic of a species’ developmental system can be inferred from its genome, and the developmental toolkits of different species can be compared. These comparisons can reveal changes in developmental logic between taxa by identifying gene losses during evolution that must result in changed pathway functioning, and similarly identify genes recruited to developmental regulatory roles in particular lineages.

Efficient generation of genomic resources for non-model species, and the inference of developmental regulatory pathways from the encoded gene sets, is now possible. The majority of the fifteen nematode genomes published to date have been from Rhabditida (Figure 1) [18-26]. The single enoplean genome sequences is from the mammalian parasite *Trichinella spiralis* (Dorylaimia; order Trichocephalida) [27]. *T. spiralis* is ovoviviparous, proper development requires intrauterine environment, and early blastomeres are extremely transparent [28] such that individual nuclei are hard to identify (E.S., unpublished observations). Hence, this species is of very limited value for light microscopical image analysis and experimental investigation correlating cell dynamics with the molecular circuitry regulating early development.

Although the genomes of many additional nematode species are being sequenced [29,30], even in this wider sampling of the phylum, Enoplea remains neglected. The enoplean *Romanomermis culicivorax* (order Mermithida within Dorylaimia) has been established in culture for decades. It infects and kills the larvae of many different mosquito species [31], and is being investigated for its potential as a biocontrol agent of malaria and other disease vectors [31,32]. *R. culicivorax* and *T. spiralis* differ fundamentally in many life-cycle and phenotypic characters. *R. culicivorax* reproduces sexually. A single female can produce more than a thousand eggs, and embryos are easily studied under laboratory conditions. They display a developmental pattern that differs markedly from *C. elegans*. As in other Enoplea [14,33] the first division is equal, and not asymmetric as in *C. elegans*. *R. culicivorax* also shows an inversion of dorso-ventral axis polarity compared to *C. elegans*, while a predominantly monoclonal fate distribution indicates fewer modifying inductions between blastomeres [33,34]. Generation of the hypodermis involves repetitive cell elements extending from posterior to anterior over the remainder of the embryo, a process distinct from that observed in *C. elegans*[34].

We here catalogue the *R. culicivorax* developmental toolkit derived from annotation of a draft genome sequence. We contrast genes and proteins identified in *R. culicivorax* and *T. spiralis* with those of *C. elegans*, and other Ecdysozoa, represented by the arthropod *Tribolium castaneum*. We conclude that major changes in the regulatory logic of development have taken place during nematode evolution, possibly as a consequence of developmental system drift, and that the model species *C. elegans* is considerably derived compared to an ecdysozoan (and possibly metazoan) ground system. However, we are still able to define conserved gene sets that may act in “phylotypic” developmental stages.

## Results and discussion

### *Romanomermis culicivorax* has a large and repetitive genome

A draft genome assembly for *R. culicivorax* was generated from 26.9 gigabases (Gb) of raw data (filtered from a total of 41 Gb sequenced; Additional file 1: Table S1). The assembly has a contig span of 267 million base pairs (Mb) and a scaffold span of 323 Mb. The 52 Mb of spanned gaps are likely inflated estimates derived from use of the SSPACE scaffolder. We do not currently have a validated independent estimate of genome size for *R. culicivorax*, but preliminary measurements with Feulgen densitometry suggest a size greater than 320 Mb (Elizabeth Martínez Salazar pers. comm.). The *R. culicivorax* genome is thus three times bigger than that of *C. elegans*, and five times that of *T. spiralis* (Table 1). The assembly is currently in 62,537 scaffolds and contigs larger than 500 bp, with an N50 of 17.6 kilobases (kb). The N50 for scaffolds larger than 10 kb is 29.9 kb, and the largest scaffold is over 200 kb. The GC content is 36%, comparable to 38% of *C. elegans* and 34% in *T. spiralis*. We identified 47% of the *R. culicivorax* genome as repetitive. To validate this estimate we applied our repeat-finding approach to previously published genomes and achieved good accordance with these data (Table 1). The non-repetitive content of the *R. culicivorax* genome is thus approximately twice that of *C. elegans* and three times that of *T. spiralis*. *T. spiralis* thus stands out as having the least complex nematode genome sequenced so far, and the contrast with *R. culicivorax* indicates that small genomes are not characteristic of Dorylaimia.

We generated 454 Sequencing transcriptome data from mixed adults, and assembled 29,095 isotigs in 22,418 isogroups, spanning 23 Mb. These are likely to be a reasonable estimate of the *R. culicivorax* transcriptome. Using BLAT [35], 21,204 of the isotigs were found to be present (with matches covering >80% of the isotig) in single contigs or scaffolds of the genome assembly, suggesting reasonable biological completeness and contiguity of the genome. We also used the CEGMA [36] approach to assess quality of the genome assembly, and found a high representation (90% partial, 75% complete) and a low proportion of duplicates (1.1 fold) (Table 2). Automated gene prediction with iterative rounds of the MAKER pipeline [37], using the transcriptome data as evidence both directly and through GenomeThreader-derived mapping, yielded a total of nearly 50,000 gene models. These were reduced to 48,171 by merging those with identities >99% using Cd-hit [38]. Within the 48,171 models, 12,026 were derived from the AUGUSTUS modeller [39] and 36,145 from SNAP. Because AUGUSTUS predictions conservatively require some external evidence (transcript mapping and/or sequence similarity to other known proteins), we regarded these as the most reliable and biologically complete. In comparison *C. elegans* has ∼22,000 genes, and *T. spiralis* has ∼16,000. The satellite model nematode *Pristionchus pacificus* has ∼27,000 genes [20]. Exons of the AUGUSTUS-predicted genes in *R. culicivorax* had a median length of 161 bp, slightly larger than those in *C. elegans* (137bp) and *T. spiralis* (128bp). Introns of the *R. culicivorax* AUGUSTUS models, with a median of 405 bp, were much larger than those of *C. elegans* (69 bp) or *T. spiralis* (283bp). The small introns observed in *C. elegans* and other rhabditid nematodes (Table 2) are thus likely to be a derived feature.

**Table 2:** Assembly and annotation statistics

**Metric**	**Result**
Contigs >100bp span	267,342,457bp
Scaffolds >500bp span	322,765,761bp
Num. contigs/scaffolds	62,537
N50 contigs/scaffolds >500bp	17,632 bp
N50 scaffolds >500bp	29,995bp
Max contig length	28,847bp
Max scaffold length	201,054bp
Mean transcript length	593bp
Mean protein length	190aa
MAKER AUGUSTUS predictions	12,026 proteins
MAKER SNAP predictions	36,145 proteins
Num. ESTs (isogroups)	22,418 ESTs
Mean EST length	330bp
80% BLAT sequence coverage	21,204 ESTs
CEGMA compl. completeness	75.40%
CEGMA Group 1 part. compl.	81.82%
CEGMA Group 2 part. compl.	91.07%
CEGMA Group 3 part. compl.	91.80%
CEGMA Group 4 part. compl.	95.38%

We annotated 1,443 tRNAs in the *R. culicivorax* genome using INFERNAL [41] and tRNAscan-SE [42], of which 382 were pseudogenes (see Additional file 1: Table S2 for details). In comparison, *T. spiralis* has 134 tRNAs of which 7 are pseudogenes, while *C. elegans* has 606 tRNAs with 36 pseudogenes [43]. Threonine (Thr) tRNAs were particularly overrepresented (676 copies), a finding echoed in the genomes of *Meloidogyne incognita* and *Meloidogyne floridensis*[24,43] and in *P. pacificus*[20]. The latter has also an overrepresentation of Arginine tRNAs [43].

We have made available the annotated *R. culicivorax* genome, with functional categorisations of predicted genes and proteins and annotation features, in a dedicated genome browser at http://romanomermis.nematod.es.

### The *R.culicivorax* gene set is more representative of Dorylaimia than *T. spiralis*

The phylogenetic placement of *R. culicivorax* makes its genome attractive for exploring the likely genetic complexity of an ancestral nematode. With *T. spiralis*, it can be used to reveal the idiosyncrasies of the several genomes available for Rhabditida. To polarise this comparison, we used the arthropod *Tribolium castaneum*, for which a high quality genome sequence is available [44]. *T. castaneum* development is considered less derived than that of the major arthropod model *Drosophila melanogaster*[45]. The OrthoMCL pipeline accurately clusters orthologous proteins, facilitating the complex task of grouping proteins that are likely to share biological function in divergent organisms [46], and performs better than approaches that simply use domain presence information or aggregative approaches such as psiBLAST [47]. We used the OrthoMCL pipeline to generate a set of protein clusters for the four species (*R. culicivorax*, *T. spiralis*, *C. elegans* and *T. castaneum*). While the large divergence between these species may obscure relationships between protein sequences, making inference of orthology problematic [48-50], the parameters used were most inclusive [50-52]. Additionally, as the *R. culicivorax* genome assembly may not be complete we based inference of absence on shared loss in both *R. culicivorax* and *T. spiralis*. Additionally, we validated inferences of absence from the OrthoMCL analyses by performing detailed sequence comparisons using BLAST+ [53] (Additional file 2).

**Figure 2:**
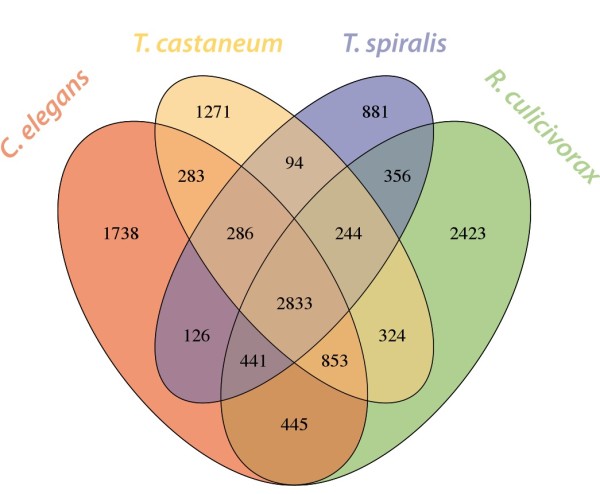
**Clusters of homologous proteins.** Shared and species-unique clusters of homologous proteins from a comparison of the proteomes of *Romanomermis culicivorax*, *Trichinella spiralis*, *Caenorhabditis elegans* and *Tribolium castaneum* using OrthoMCL.

We identified 3,274 clusters that contained protein representatives from all three nematodes, and 2,833 of these also contained at least one *T. castaneum* representative (Figure 2). These 2,833 clusters represent a conserved ecdysozoan (and possibly metazoan) core proteome. Many clusters had *T. castaneum* members, and members from some but not all of the three nematodes, representing candidate examples of loss in one or more nematode lineages of ancient proteins. For example, we identified clusters containing proteins from only one of the nematode species. *T. spiralis* had the lowest number of these (975), while *C. elegans* and *R. culicivorax* each had over two thousand. Interestingly, of the 2,747 clusters with only *R. culicivorax* proteins from Nematoda, 324 included *T. castaneum* orthologues, wheras *C. elegans* only shared 283 clusters uniquely with the beetle. *T. spiralis* has lost more of these phylogenetically ancient genes than has either *R. culicivorax* or *C. elegans*. *T. spiralis* and *C. elegans* shared only 412 clusters exclusive of *R. culicivorax* members, while *R. culicivorax* and *C. elegans* shared about 1300 clusters exclusive of *T. spiralis*. Despite their phylogenetic affinity, *R. culicivorax* and *T. spiralis* only shared 600 clusters exclusive of *C. elegans* (Figure 2). We suggest that *T. spiralis* genome is not typical of dorylaims. In comparison to other nematodes it is smaller, has fewer genes overall, and has fewer phylogenetically ancient genes. This is congruent with the previously reported loss of proteins with metabolic function in *T. spiralis*[27]. This reduction in genetic complexity could be due to evolutionary pressures following acquisition of a lifestyle that lacks a free-living stage. Many parasitic and endosymbiotic prokaryotes and eukaryotes have reduced genome sizes, though this is not an absolute rule [54].

Clusters containing only *R. culicivorax* and *T. spiralis* proteins might identify functions distinct to these dorylaim nematodes. In the 461 *T. spiralis* and 806 *R. culicivorax* proteins in these clusters, a total of 65 GO terms were found to be overrepresented (single test p <0.05 by Fisher’s exact test). While *C. elegans* has a reduced ability to methylate DNA [55], we found four methylation-associated GO terms among the 64 overrepresented. We also detected significant enrichment (single test p <0.05) for GO terms describing chromatin and DNA methylation functions in the set of *R. culicivorax* proteins that lacked homologues in *C. elegans* (see Additional file 3). Important roles for methylation and changes in methylation patterns during *T. spiralis* development have been inferred from transcriptional profiling [56]. Methylation is important for the silencing of transposable elements [57,58] and could play a crucial role in the highly repetitive *R. culicivorax* genome.

The clusters that contained *R. culicivorax*, *T. spiralis* and *T. castaneum* proteins but no *C. elegans* orthologues might contain proteins involved in core ecdysozoan processes lost in *C. elegans*. In these clusters we identified 40 GO terms overrepresented (single test p <0.05) compared to the *C. elegans* proteome (see Additional file 3). Some of these GO terms were linked to chromatin remodelling and methylation (e.g. Ino80 complex, histone arginine methylation). Other overrepresented GO terms were related to cell signalling (the Wnt receptor pathway; the C. elegans Wnt signalling system is distinct from other metazoa [59]), and ecdysone receptor holocomplex (potentially a basic ecdysozoan function [60]).

### The genetic background of development in *R. culicivorax* and *T. spiralis* differs markedly from that of *C. elegans*

In a recent multi-species developmental time course expression analysis within several *Caenorhabditis* species, conserved sets of genes were found to have conserved patterns of differential expression in discrete phases in the timeline from zygote to the hatching larva [61].

**Figure 3:**
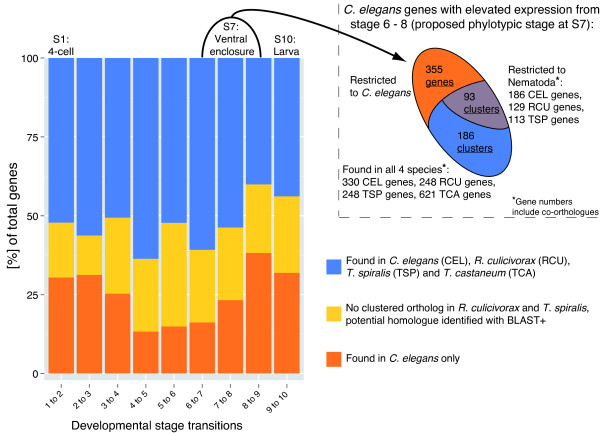
**Many genes that are developmentally important in *Caenorhabditis elegans *were not present in *Romanomermis culicivorax *or *Trichinella spiralis*.***R. culicivorax* and *T. spiralis* orthologues of the 1,725 genes identified as important in embryogenesis in an analysis of gene expression in *Caenorhabditis* species [61] were sought. For each embryonic stage (1-10) in *C. elegans* we calculated the proportion of these genes that were apparently unique to the genus *Caenorhabditis*. The inset depicts numbers of two sets of genes and corresponding clusters that could play a role in a phylotypic stage of Nematoda or all Ecdysozoa, respectively.

Nearly half (845) of these 1725 conserved, differentially expressed *C. elegans* proteins were not clustered with *R. culicivorax* or *T. spiralis* proteins using OrthoMCL. We were unable to identify any sequence similarity for 450 of these *C. elegans* proteins, while 395 had only marginal similarities insufficent for OrthoMCL clustering. Eighteen of these 395 are members of *C. elegans* nuclear hormone receptor subfamilies, 5 are innexin type gap-junction proteins, 6 are TWiK potassium channel proteins and 5 are acetylcholine receptor proteins. These protein families are particularly diverse and expanded in *C. elegans*[62-65] and we suggest that they represent rapidly evolved, divergent duplications within the lineage leading to *C. elegans*. The proportion of *Caenorhabditis*-restricted genes across the developmental time course examined by Levin et al. [61] varied from 36% to 60% (Figure 3 and Additional file 4). Thus a surprisingly high proportion of *Caenorhabditis* genes with conserved expression during embryogenesis appear to be unique to the genus or are so divergent that we could not detect possible orthologues in the dorylaims. The pattern of higher retention of conserved genes in *R. culicivorax* compared to *T. spiralis* was also evident in these conserved-expression developmental genes, as 238 had *R. culicivorax* orthologues but lacked a *T. spiralis* orthologues. Given the conservatism of body plan evolution in nematodes, these dramatic genetic differences suggest extensive, largely phenotypically “silent” changes in the genetic programmes orchestrating nematode development.

### Core developmental pathways differ between nematodes

There are important differences in cell behaviour during early embryogenesis between *R. culicivorax* and *C. elegans*[33,34]. We used the genomic data to follow up on some of the striking contrasts between the dorylaim and the rhabditid patterns of development: establishment of primary axis polarity, segregation of maternal message within the early embryo, hypodermis formation, the vulval specification pathways, epigenetic pathways (especially DNA methylation), sex determination and light sensing (see Additional file 1).

The mechanisms of sex determination differ considerably among animals and it has been claimed to be one of the developmental programs most influenced by developmental system drift [16]. Divergence in sex determination pathways is thus not unexpected. While sex is determined by X to autosome ratio in *C. elegans*[66], sex ratios in *R. culicivorax* are likely to be environmentally determined through in-host nematode density [67]. Environmental sex determination is found in many nematode taxa, including Strongyloididae and Meloidogyninae (both Tylenchina), taxa more closely related to *C. elegans*. In *C. elegans*, the X to autosome ratio is read by the master switch XOL-1 [68], which acts through the three *sdc* genes [69-71] to regulate the secretion of HER-1, a ligand for the TRA-2 receptor [72-74]. TRA-2 in turn negatively regulates a complex of *fem* genes, which regulates nuclear translocation of TRA-1, the final shared step in the pathway that switches between male and hermaphrodite systems. No credible homologues of XOL-1, SDC-1, SDC-2, SDC-3, HER-1 or TRA-2 in either *T. spiralis* or *R. culicivorax* were detected through OrthoMCL and re-confirmation with BLAST+ (Table 3; Additional file 2), and thus these species are unlikely to use the HER-1 – TRA-2 ligand-receptor system to coordinate sexual differentiation.

**Table 3:** **Presence and absence of selected**^**∗**^ ***C. elegans* proteins in Dorylaimia**

**Protein**	***T. spiralis***	***R. culicivorax***
**Early asymmetry**		
CDC-42	+	+
PKC-3	+	+
GPR-1	-	-
GPR-2	-	-
PAR-2	-	-
PAR-6	+	+
MES-6	+	+
MES-3	-	-
MES-4	-	-
GFL-1	+	+
LET-70	+	+
**Axis formation**		
NUM-1	+	+
ZIM-1	-	-
MES-2	-	-
POS-1	-	-
SMA-6	+	+
SET-2	-	-
UBC-18	+	+
LET-99	-	-
OOC-3	-	-
OOC-5	+	+
GPA-16	+	+
PAR-5	-	-
ATX-2	-	-
MEX-5	-	-
MEX-6	-	-
UNC-120	-	-
NOS-2	-	-
OMA-1	-	-
RME-2	+	+
SPN-4	-	-
**Sex determination**		
XOL-1	-	-
HER-1	-	-
SEX-1	+	+
FOX-1	+	+
SDC-1	-	-
SDC-2	-	-
SDC-3	-	-
TRA-2	-	-
FEM-1	+	+
FEM-2	+	+
AFF-1	-	-
BAR-1	-	-
CEH-2	-	-
CEH-27	-	-
GRL-15	-	-
INX-5	-	-
LIN-1	-	-
PEB-1	-	-
ELT-3	-	-
ELT-1	+	+
SMA-3	-	-
SMA-5	-	-

^*^For additional proteins see Additional files 2 and 4.

**Figure 4:**
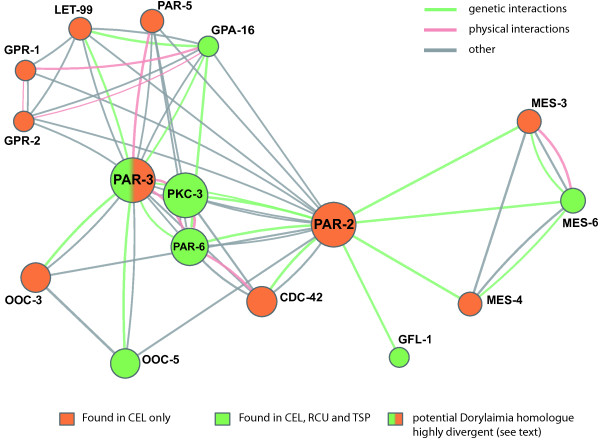
**The network of proteins interacting with PAR-2 and PAR-3 in *Caenorhabditis elegans *and their orthologues in *Romanomermis culicivorax *and *Trichinella spiralis*.** The network cartoon is based on the core polarity pathway extracted from WormBase, derived from both genetic and physical interactions. PAR-2 was missing from the dorylaim nematodes, as were the directly connected MES-3 and MES-4 genes. The *R. culicivorax* PAR-3-like protein was not retrieved as an orthologue of *C. elegans* and *T. spiralis* PAR-3 proteins, but was identified employing sensitive sequence similarity search. See Table 3 for additional proteins interacting with PAR proteins and their presence-absence patterns.

Other developmental processes are however more conserved between metazoan taxa. In *C. elegans* and many other animals *par* genes are essential for cell polarisation [75]. Polarised distribution of PAR proteins results in the restriction of mitotic spindle rotation to the germline cell in the *C. elegans* two-cell stage [76-78]. This rotation is not observed in *R. culicivorax*[33]. The division pattern of *C. elegans* mutants lacking *par-2* and *par-3* genes resembles that of the early *R. culicivorax* embryo [33,79]. The *par-2* gene was absent from both *R. culicivorax* and *T. spiralis* (Figure 4; Table 3). Additionally, no orthologues for the *par-2*-interacting genes *let-99*, *gpr-1* or *gpr-2*, required for proper embryonic spindle orientation in *C. elegans*[80], were identified in the dorylaims using OrthoMCL clustering or sensitive BLAST+ searches. Although we identified a protein with weak similarity to *par-3* in *R. culicivorax*, this was so divergent from *C. elegans*, *T. castaneum* and *T. spiralispar-3* that it was not clustered in our analysis. In *D. melanogaster* a *par-3* orthologue, *bazooka*, functions in anterior-posterior axis formation [81], but *par-2* is absent from the fly. Thus, we hypothesise that the PAR-3/PAR-2 system for regulating spindle positioning evolved within the lineage leading to the genus *Caenorhabditis*. If the divergent *par-3*-like gene in *R. culicivorax* is involved in axis formation, it probably interacts with different partner proteins.

**Figure 5:**
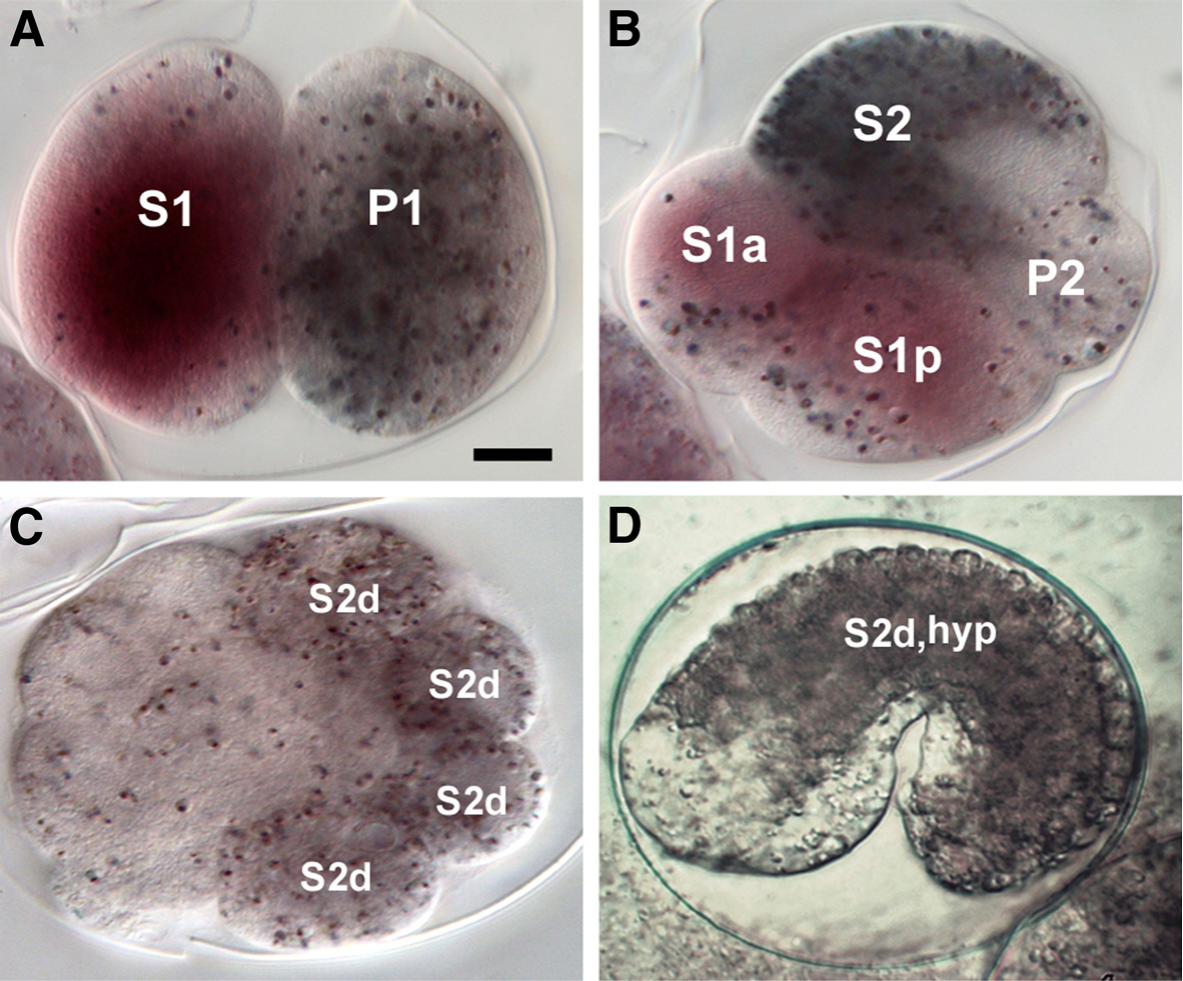
***In situ *hybridisation mapping of *mex-3 *mRNA distribution in early embryos of *Romanomermis culicivorax*.** We used the *R. culicivoraxmex-3* gene to prove application of the *in situ* technique in this species and investigated the segregation patterns of segregation of this maternal RNA in early development. The *R. culicivoraxmex-3* expression pattern is similar to that of *C. elegans*[83]. *R. culicivorax* embryos contain dark pigment granules that are asymmetrically segregated in development. **(A)** At the 2-cell stage, maternal *mex-3* mRNA is detected in the S1 blastomere. The cytoplasmic pigment granules are predominantly in the P1 blastomere. **(B)** At the 4-cell stage, *mex-3* mRNA is detected in daughters of the anterior S1 cell. Cytoplasmic pigment granules are predominantly in the S2 blastomere. **(C)** At a later stage (>20 cells), *mex-3* mRNA is absent. The pigment granules are found in descendants of S2 (S2d). **(D)** During early morphogenesis, the pigment granules are found in S2 descendants forming hypodermis, (S2d, hyp). **(A-C)** fixed embryos; **(D)** live embryo. Scale bar 10 *μ*m. Orientation: anterior left.

Once polarity has been established in the early *C. elegans* embryo, many maternal messages are differentially segregated into anterior or posterior blastomeres [78,82]. MEX-3 is an RNA-binding protein translated from maternally-provisioned mRNAs found predominantly in early anterior blastomeres [83,84]. We identified a highly divergent MEX-3 orthologue in *R. culicivorax*, but no orthologue in *T. spiralis*. We explored embryonic expression of *mex-3* in *R. culicivorax* embryos using *in situ* hybridisation (Figure 5). In the fertilized egg the *mex-3* mRNA is initially equally distributed. Prior to first cleavage it is segregated to the anterior pole and thus becomes essentially restricted to the somatic S1 blastomere (for nomenclature, see [14]). With the division of S1 it is localized to both daughter cells. After the 4-cell stage the signal disappears gradually. This expression pattern is similar to that of *C. elegansmex-3*, affirming that the *R. culicivorax* gene is likely to be an orthologue retaining similar functions. However, despite the presence, and apparent conservation of the *mex-3* expression pattern, we were unable to identify other interacting partners of the *C. elegans* MEX-3 protein, such as MEX-5, MEX-6 and SPN-4 in either dorylaim species. While MEX-5 and MEX-6 are important for controlled MEX-3 expression in *C. elegans*[85], the apparent absence of SPN-4 in *R. culicivorax* and *T. spiralis* is particularly intriguing. SPN-4 links embryonic polarity conferred by the *par* genes and partners to cell fate specification through maternally deposited mRNAs and proteins [86,87]. Our findings suggest that the core regulatory logic of the early control of axis formation and cell fate specification must differ significantly between the dorylaim species and *C. elegans*.

The hypodermis in *C. elegans* is derived from specific descendants of the anterior and posterior founder cells [88]. In contrast, in *R. culicivorax* hypodermis is derived from descendants of a single cell [34]. Several *C. elegans* genes expressed in the hypodermis or associated with hypodermal development were absent from *R. culicivorax* and *T. spiralis* (see Table 3 and Additional file 3). For example the GATA-like transcription factors ELT-1 and ELT-3 act redundantly in *C. elegans*[89]. ELT-3 was absent from the dorylaim species, but ELT-1 was conserved in *R. culicivorax*, *T. spiralis* and *T. castaneum*. Thus, ELT-3 appears to be an innovation in the rhabditid lineage, suggesting changes of interaction complexity during nematode evolution.

In *C. elegans*, vulva formation is highly dependent on initial inductive signals from the anchor cell that activate a complex gene regulatory network, which drives tissue specific cell division and differentiation. The evolutionary plasticity of this system has been explored in rhabditid nematodes, revealing the changing relative importance of cell-cell interactions, inductions, and lineage-autonomous specifications [90,91]. The signal transduction pathways include a RTK/RAS/MAPK cascade, activated by EGF- and wnt-signalling [92]. Among the downstream targets in *C. elegans* are for example LIN-1 and the *β*-catenin BAR-1, which in turn regulates the HOX-5 orthologue LIN-39 [93-95]. These important regulators of vulva development are completely absent from the genomes of *R. culicivorax* and *T. spiralis* (Table 3 and Additional file 2). We identified a *R. culicivorax* protein with low similarity to *C. elegans* BAR-1 (24% sequence identity). However, this protein is not clustered with other dorylaim proteins, and appears to be either a duplication of the *β*-catenin ortholog HMP-2 or another armadillo repeat-containing protein rather than an orthologue of BAR-1 (see Additional file 5). These shared patterns of absence again suggest that similar morphological structures can be generated with very different genetic underpinnings. Vulva formation in the dorylaims may be regulated without the BAR-1 – LIN-39 interaction, as observed in *P. pacificus*[96]. In *C. elegans* Hox gene expression is cell-lineage dependent [97,98], organised so that the cells that express specific Hox genes are clustered along the anterior-posterior axis (see e.g. [99]). It will be informative to test whether in *R. culicivorax* and other non-rhabditid nematodes Hox genes act in an axis position-dependent, but cell lineage-independent manner, as observed in many other animals, notably arthropods [100,101]. Epigenetic regulation is key to developmental processes in many animals, but its roles in *C. elegans* are more muted (see above). Notably *C. elegans* is depleted for chromatin re-modelling genes of the Polycomb and Trithorax groups [102]. It is intriguing that we found orthologues of *T. castaneumpleiohomeotic* in *R. culicivorax* and *T. spiralis*, and orthologs of *T. castaneumtrithorax* and *Sex comb on midleg* (*Scm*) in *R. culicivorax*. This suggests that dorylaim chromatin restructuring mechanisms may be more arthropod-like than in *C. elegans*. The presence of an intact methylation machinery and conserved chromatin re-modelling factors opens the prospects for a role for epigenetic modification in developmental regulation of dorylaim nematodes.

### Defining a set of potential phylotypic stage genes

While the examples above demonstrate considerable developmental system drift in Nematoda, we also identified many sets of orthologous proteins conserved between Dorylaimia and *C. elegans*. We asked if these could be correlated with functions in distinct developmental phases with a conserved phenotype. Shortly before the start of morphogenesis, at the point of ventral enclosure, nematode embryos from Chromadorea and Enoplea share a similar morphology [14]. Levin et al. [61] found that in five *Caenorhabditis* species a distinct set of genes had elevated expression around the ventral enclosure stage (their stage 7) (Figure 3) and proposed that this constitutes a “phylotypic stage" for nematodes. We used *T. spiralis* and *R. culicivorax* gene sets to refine and restrict this set of phylotypic stage genes. Of the 834 *C. elegans* genes with elevated expression between stages 6 to 8 [61], 355 had no orthologue in *R. culicivorax*, *T. spiralis* or *T. castaneum*. The remaining 479 phylotypic stage candidates from *C. elegans* were present in 279 of our OrthoMCL clusters. Of these clusters 93 were nematode-restricted containing 186 *C. elegans* proteins grouped with 129 *R. culicivorax* and 113 *T. spiralis* homologues. The remaining 186 clusters were part of the conserved ecdysozoan core proteome (see above) and contained 330 *C. elegans* proteins together with 248 *R. culicivorax*, 248 *T. spiralis* and 621 *T. castaneum* proteins (Figure 3; The total number of *C. elegans* candidates is larger than 479 due to the inclusion of co-orthologues in this species). In the set of phylotypic stage genes identified by Levin et al. [61] are proteins functioning in processes such as muscle and neuron formation, signalling between cells, and morphogenesis. This pattern was retained in the conserved clusters (see Additional file 5). Although time-resolved expression data will be needed to confirm the activity of these genes in developmental stages of *R. culicivorax*, their retention in the Dorylaimia supports their general importance. We can now sub-classify the set of conserved proteins expressed at the potential nematode phylotypic stage. A first, nematode-restricted set includes many proteins that are important for cuticle formation (e.g. collagen proteins) and some hedgehog-like proteins, expressed in the *C. elegans* hypodermis [103]. As cuticle formation follows ventral enclosure in nematodes, these proteins may be involved in this nematode-specific function. The second set, comprising clusters conserved between the nematodes and *T. castaneum*, contains many important developmental transcription factors, such as the Hox gene *mab-5*, other homeobox genes, and helix-loop-helix and C2H2-type zinc finger transcription factors. This second set may represent a genetic backbone driving formation of phylotypic stage in diverse animal taxa, in accordance with the recent extension of the concept to Metazoa [104-106].

## Conclusions

To be useful as a contrasting system to the canonical *C. elegans* model, any nematode species must be accessible to both descriptive and manipulative investigation. The reference genome for *R. culicivorax* lays bare the core machinery available for developmental regulation, and we have demonstrated that *in situ* hybridisation approaches are feasible for this species. Along with the long established, robust laboratory cultures, this makes *R. culicivorax* an attractive and tractable alternative model for understanding the evolutionary dynamics of nematode development. By combining the *R. culicivorax* genome with that of *T. spiralis*, we have been able to explore the molecular diversity of Dorylaimia, and provide robust contrasts with the intensively studied Rhabditida. Particularly surprising are the differences between *R. culicivorax* and *T. spiralis*. The *R. culicivorax* genome is much larger than that of *T. spiralis*, and contained a high proportion of repetitive sequence, including many transposable elements. Despite the phylogenetic and lifestyle affinities between the two dorylaims compared to *C. elegans*, the *R. culicivorax* genome retained many more genes in common with *C. elegans* than did *T. spiralis*. We suggest that *T. spiralis* may be an atypical representative of dorylaim nematodes, perhaps due to its highly derived life cycle.

Our analyses identified many genes apparently absent from the dorylaim genomes, despite relaxed analysis parameters. In particular, for genes identified as critical to *C. elegans* development but apparently absent from the dorylaims, we were unable to identify credible orthologues using sensitive search strategies. In this phylum-spanning comparison, inferences of gene orthology can be obscured by levels of divergence. In addition, the gene family birth rate in the chromadorean lineage leading to *C. elegans* is high [25,27], and therefore *C. elegans* was expected to have many genes absent from the dorylaim species. Thus, we might not have found a *R. culicivorax* orthologue for a specific gene for three reasons: it may have arisen in the branch leading to *C. elegans*; its sequence divergence may be too great to permit clustering with potential homologs; or it was not assembled in the draft dorylaim genomes. The case of *C. elegans* PAR-3 and *D. melanogasterbazooka* illustrate some of these difficulties: the possible *R. culicivorax* orthologue was highly divergent. Whether or not we have been able to identify all the orthologues of the key *C. elegans* genes present in the *R. culicivorax* and *T. spiralis* genomes, the absence of an identified orthologue maximally implies loss from the genome, and minimally implies significant sequence divergence. In the latter case this would most likely cause changes in the networks and pathways in which genes interact to deliver biological function.

Between the model nematode *C. elegans* and arthropod models such as *T. castaneum* many key mechanisms governing early cell patterning are divergent [76]. Our data strongly support the view that major variation also exists within Nematoda. *T. spiralis* and *R. culicivorax* both lack orthologues of genes involved in core developmental processes in *C. elegans*, and many of these *C. elegans* genes appear to be restricted to the Rhabditida. It is thus doubtful that these processes are regulated by same molecular interactions across the phylum. We suggest that developmental system drift has played a major role in nematode evolution. The phenotypic conservatism associated with the vermiform morphology of nematodes [107] has fostered unjustified expectations concerning the conservation of genetic programmes that determine these morphologies. Despite this divergence in developmental systems, we were able to define two sets of conserved genes possibly active in a taxon-specific phase of ventral enclosure and cuticle formation in Nematoda, and in a potential phylotypic stage of Ecdysozoa. The advent of robust, affordable and rapid genome sequencing also opens the vista of large-scale comparative genomics of development across the phylum Nematoda [29] to better understand the diversity of the phylum and also place the remarkable *C. elegans* model in context of its peers. It will next be necessary to extend these studies to a broader sampling of developmental pathway genes from a wider and representative sampling of nematode genomes across the full diversity of the phylum. We have highlighted a few of the possible avenues a research programme could follow: early axis formation and polarisation, the specification of hypodermis, sex determination, vulva formation, the roles of epigenetic processes in developmental regulation and the confirmation of potential “phylotypic stage genes” with expression analysis in *R. culicivorax*.

## Methods

### Sequencing and genome assembly

Genomic DNA was extracted from several hundred, mixed-sex, adult *R. culicivorax* specimens from a culture first established in Ed Platzer’s laboratory in Riverside, California. Illumina paired end and mate pair sequencing with libraries of varying insert sizes, and Roche 454 single end sequencing, was performed at the Cologne Center for Genomics (CCG: http://www.ccg.uni-koeln.de). A Roche 454 dataset of transcriptome reads from cDNA synthesised from mixed developmental stages and sexes was also generated (see Additional file 1: Table S1 for details of data generated).

The quality of the raw data was assessed with FastQC (v.0.9; http://www.bioinformatics.babraham.ac.uk/projects/fastqc/). Adapter sequences and low quality data were trimmed from the Illumina paired end data with custom scripts (see http://github.com/sujaikumar/assemblage) and from the mate pair libraries with Cutadapt (v.1.0) [108]. We constructed a preliminary genome assembly, with relaxed insert size parameters, from the paired end Illumina libraries with the de-novo-assemble option of the clcAssemblyCell (v.4.03b) [109]. We validated the actual insert sizes of our libraries by mapping back the reads to this preliminary assembly using clcAssemblyCell. The preliminary assembly was also used to screen out bacterial and other contaminant data [110]. The transcriptome data were assembled with Roche GSAssembler (Newbler; version 2.5). For the production assembly, we explored assembly parameters using different mixes of our data, evaluating each for total span, maximal contig lengths, N50, number of contigs, representation of the transcriptome, and conserved eukaryotic gene content (using the CEGMA pipeline v.2.1 [36]). The most promising assembly was scaffolded with the filtered Illumina mate pair read sets using SSPACE (v.1.2) [111]. As our genomic DNA derived from a population of nematodes of unknown genetic diversity, we removed short contigs that mapped entirely within larger ones using Cd-hit (v.4.5.7) [38] at a 95% cutoff. A final round of superscaffolding was performed, linking scaffolds that had logically consistent matches to the transcriptome data based on BLAT [35] hits and processed with SCUBAT (B. Elsworth, pers. comm.; http://github.com/elswob/SCUBAT). The final genome assembly was again assessed for completeness by assessing the mapping of the transcriptome contigs and with the CEGMA pipeline [36].

### Genome annotation

RepeatMasker (v.3.3.0) [112,113], RepeatFinder [114] and RepeatModeler (v.1.0.5; http://www.repeatmasker.org/RepeatModeler.html; combining RECON (v.1.07) [115] and RepeatScout (v.1.05) [116]), were used to identify known and novel repetitive elements in the *R. culicivorax* genome. We employed the MAKER pipeline[37] to find genes in the *R. culicivorax* genome assembly. In a first pass, the SNAP gene predictor included in MAKER was trained with a CEGMA [36] derived output of predicted highly conserved genes. As additional evidence we included the transcriptome assembly and a set of approximately 15,000 conserved nematode proteins derived from the NEMBASE4 database[117] (recalculated by J. Parkinson; pers. comm.). In the second, definitive, pass we used the gene set derived from this first MAKER iteration to train AUGUSTUS [39] inside the MAKER pipeline for a second run, also including evidence from transcriptome to genome mapping obtained with GenomeThreader [118]. Codon usage in *R. culicivorax*, *T. spiralis*, and *C. elegans* was calculated using INCA (v2.1) [119]. Results were then compared to data from [120] (see Additional files 1 and 6).

We used Blast2GO (Blast2GO4Pipe, v.2.5, January 2012 database issue) [121] to annotate the gene set with Gene Ontology terms [122], based on BLAST matches with expect values less than 1e ^-5^ to the UniProt/SwissProt database (March 2012 snapshot), and domain annotations derived from the InterPro database [123]. Comparison of annotations between three nematode species (*R. culicivorax*, *C. elegans*, and *T. spiralis*) and, as a reference outgroup, the holometabolous coleopteran arthropod *Tribolium castaneum*, was based on GO Slim data retrieved with Blast2GO. RNA genes were predicted using INFERNAL (v.1.0.2)[41] and the Rfam database [124], and tRNAscan-SE (v.1.3.1) [42].

### Orthology screen

We inferred clusters of orthologous proteins between *R. culicivorax*, *T. spiralis*, and *C. elegans*, and the beetle *T. castaneum* using OrthoMCL (v.2.0.3) [125]. *T. spiralis*, *C. elegans* and *T. castaneum* protein sets were downloaded from NCBI and WormBase (see Additional file 1: Table S3) and redundancy screened with Cd-hit at the 99% threshold. We selected an inflation parameter of 1.5 for MCL clustering (based on [126,127]) within OrthoMCL to generate an inclusive clusterings in our analysis likely to contain even highly diverged representatives from the four species. In analyses of selected developmental genes, clusters were manually validated using NCBI-BLAST+ [53]. We affirmed the uniqueness of *C. elegans* proteins identified as lacking homologues in the enoplean nematodes by comparing them to the *R. culicivorax* proteome using BLAST. Those with no significant matches at all (all matches with E-values > 1e ^-5^) were classified as confirmed absent. Those having matches with E-values < 1e ^-5^ were investigated further by surveying the cluster memberships of the *R. culicivorax* matches. If the *R. culicivorax* protein was found to cluster with a different *C. elegans* protein, the uniqueness to *C. elegans* was again confirmed. If the *R. culicivorax* protein did not cluster with an alternative *C. elegans* protein, we reviewed the BLAST statistics (E-value, identity and sequence coverage) of the match and searched the GenBank non redundant protein database for additional evidence of possible orthology. Only if these tests yielded no indication of direct orthology was the *C. elegans* protein designated absent from the enoplean set. Further details of the process are given in Additional file 5.

We identified the protein sequences of 1,725 genes differentially expressed in *C. elegans* developmental stages [61] and selected, using our OrthoMCL clustering, those apparently lacking orthologues in *R. culicivorax* and *T. spiralis* (verified as above). Using Wormbase (http://www.wormbase.org, release WS233) we surveyed the *C. elegans*-restricted genes for their experimentally-defined roles in development.

Custom Perl scripts were used to group orthoMCL clusters on the basis of species membership patterns. The sets of clusters that contained (i) both *T. spiralis* and *R. culicivorax* members but no *C. elegans* members and (ii) *T. spiralis* and *R. culicivorax* and *T. castaneum* members but no *C. elegans* members were surveyed for GO annotations enriched in comparison to the whole *C. elegans* proteome (sets i and ii) and the *T. castaneum* proteome (set i), conducting Fisher’s exact test as implemented in Blast2GO. Due to the small size of both sets compared to the large reference set, p-values could not be corrected for multiple testing. To improve annotation reliability, these proteins were recompared (using BLAST) to the UniProt/SwissProt database and run through the Blast2GO pipeline as described above.

### Whole-mount in situ hybridization

For in situ hybridisation we modified the freeze-crack procedure described previously for *C. elegans*[128] and revised by Maduro et al. (2007; http://www.faculty.ucr.edu/~mmaduro/resources.htm). In particular, to achieve reliable penetration of the durable *R. culicivorax* egg envelopes we initially partly removed the protective layer by incubation in alkaline bleach solution (see [33]). Digoxygenine-labeled sense and antisense RNA probes were generated from linearized pBs vectors (Stratagene, La Jolla, USA) containing a 400 bp fragment of *R. culicivoraxmex-3* via run off *in vitro* transcription with T7 or T3 RNA-polymerase according to the manufacturer’s protocol (Roche, Mannheim, Germany). The concentration of the labelled probes was about 300 *ng*×*ml*^-1^.

## Competing interests

The authors declare that they have no competing interests.

## Authors’ contributions

PHS conceived study, assembled and annotated the genome, conducted analyses and wrote paper; MK conceived study, conducted analyses and wrote part of the paper; CK conceived part of the study, conducted analyses on developmental expression set and wrote part of the paper; GDK helped with genome assembly and annotation; SK helped with genome assembly and wrote/provided Perl scripts; JIRC analysed MEX-3 dataset; NAN analysed PAR dataset; DS analysed SEX determination dataset; KM conducted RNA sequencing and initial EST assembly; PH performed preparative laboratory experiments and conceived sequencing strategy; JA conceived sequencing strategy and conducted genome sequencing; PF helped with initial genome pre-assembly; PN initiated study and conceived sequencing strategy; WKT conceived parts of study; MLB conceived study and wrote paper; ES initiated and conceived study and wrote paper. All authors read and approved the final manuscript.

## Supplementary Material

Additional file 1 Supplementary data figures and tables. Click here for file

Additional file 2 **Analysis of OrthoMCL output by BLAST+.** BLAST+ results for specific *C. elegans* proteins not found in a cluster with Dorylaimia proteins. Click here for file

Additional file 3 **Fisher’s exact test data.** GO terms enriched in a set of protein clusters shared between Dorylaimia in comparison to (i) *C. elegans* and (ii) *T. castaneum* proteomes. Click here for file

Additional file 4 **Levin data.** Genes identified as being differentially expressed in *Caenorhabditis* development by Levin et al. [61]. Click here for file

Additional file 5 **Analysis of Phylotypic stage genes.***C. elegans* orthologues of genes possibly acting in (i) a potential nematode specific phylotypic stage and (ii) a metazoan phylotypic stage. Click here for file

Additional file 6 **Codon usage in *R. culicivorax*.** Codon usage data. Click here for file

## Accession numbers

Raw genome and transcriptome sequence data reported in this manuscript have been deposited in the ERA under accession ERP002111 (http://www.ebi.ac.uk/ena/data/view/ERP002111), and assembled genomic contigs deposited in the ENA INSDC database under accession numbers CAQS01000001-CAQS01062537. Annotation information and additional data are available through (http://romanomermis.nematod.es).
