# Supplementary material for: The genome of Romanomermis culicivorax: revealing fundamental changes in the core developmental genetic toolkit in Nematoda
Source: BMC Genomics. 2013 Dec 27;14:923. doi: 10.1186/1471-2164-14-923 (PMC3890508; doi:10.1186/1471-2164-14-923)
Supplement: Additional file 1 — Supplementary data figures and tables. [file 1471-2164-14-923-S1.pdf]

Supplementary Data, Figures and Tables

Light sensing machinery in R. culicivorax

Light sensing with and without eyes has been described in other mermithids [1,2]. Although *R. culicivorax* has no structurally visible eyes it appears likely that invasion of the mosquito host [3] on the surface of the water body, and migration of emerged nematodes back to the substrate to mate and deposit eggs [4] is due to phototactic behavior. Preliminary experiments with *R. culicivorax* give support to this view (J. Burr, pers. comm.), but the underlying physiology has not been explored in this nematode.

In *Mermis nigrescens*, a close relative of *R. culicivorax*, a directional light sensing organ is found in the anterior pharynx, where a cylinder of light-shadowing cells packed with a nematode hemoglobin shades a central photoreceptor [1, 5, 6]. Although globins are a large and diverse gene family in Nematoda [7], it is intriguing that we find one GO - ID (GO:0019825), which has considerable more proteins associated in *R. culicivorax* than in any of the other species analysed (see Supplementary Figure 1), containing several proteins with best BLAST hits to bonafide globins and hemoglobins. Pigment granules are segregated into the hypodermis of this species (see Figure 5) and may also have a light-shadowing function [8].

Interestingly, the GO term "cellular pigment accumulation" in the set of *R. culicivorax* proteins that had homologues with *T. spiralis* and *T. castaneum*, but not with *C. elegans*. The protein associated with this GO term was most similar to *Xenopus* SHROOM2 protein, which is expressed in the eye of the frog [9]. We identified several GO terms associated with photoreceptor development and light sensing (see Supplementary file 2) in *R. culicivorax* proteins in comparison to *C. elegans* and *T. castaneum* proteomes (in the set of *R. culicivorax* proteins without homologues in these species). Two especially intriguing GO terms were "phototaxis" and "energy taxis". Proteins associated with these GO terms had BLAST similarities to COUP transcription factors, which in the mouse have been associated with cell fate determination in the eye [10].

In addition, we identified a candidate opsin in *R. culicivorax*. The gene is partially supported by EST data, and could generate a 313 amino acid protein with identities of 26% to the *Bos taurus* (accession NP_776991) and *Didelphis aurita* (ABC75817) long-wave-sensitive opsins. These findings might allow to explore the genomic and physiological background of light sensing in *R. culicivorax* in future studies.

Supplementary Table 1 - Obtained Sequencing Libraries

2nd Generation Sequencing libraries used in *R. culicivorax* genome assembly and scaffolding. Three paired end (pe) libraries of different insert size were generated and used for contig assembly with Illumina technology. Due to the large genome size the Roche 454 single end (se) long reads were not useful in the final assembly. Scaffolding of the genome was done with Illumina mate pair (mp) reads. See main Methods section for programs used and applied strategy in read cleaning, contig building and scaffolding of the genome.

*Roche 454 library was cleaned by Sequencing Center.

**insert size (bp)**	**read length (bp)**	**technology**	**paired**	**number of reads**	**post cleaning**	**mean size post cleaning**	**no. bases post cleaning**	**used in final assembly**
150	100	Illumina pe	yes	202,136,864	160,963,290	95.1 (sd 14.4)	14,343,714,370	no
200	100	Illumina pe	yes	161,439,782	150,318,628	91.4 (sd 9.5)	13,744,221,880	yes
300	100	Illumina pe	yes	82,655,654	77,136,338	91.4 (sd 9.5)	7,049,762,982	yes
1750	36	Illumina mp	yes	122,367,532	98,168,674	32.6 (sd 8.0)	3,199,261,718	yes
2000	36	Illumina mp	yes	120,076,148	94,892,075	31.3 (sd 9.0)	2,968,120,350	yes
n/a	450	Roche 454 se	no	n/a*	4,060,548	429.4 (sd 144.1)	403,103,035	no

Supplementary Table 2 - tRNAs annotated in the R. culicivorax genome.
The *R. culicivorax* genome has a large number of Threonine tRNAs (see main text). Anticodons without identified tRNAs are not displayed.

Isotype	Count	Anticodon Counts
Alanine	30	AGC: 4, GGC: 3, CGC: 2, TGC: 21
Glycine	16	GCC: 12, CCC: 1, TCC: 3
Proline	20	AGG: 7, GGG: 3, CGG: 6, TGG: 4
Threonine	676	AGT: 29, GGT: 155, CGT: 25, TGT: 467
Valine	13	AAC: 8, CAC: 3, TAC: 2
Serine	66	AGA: 9, GGA: 16, CGA: 7, TGA: 28, ACT: 1, GCT: 5
Arginine	26	ACG: 7, CCG: 1, TCG: 4, CCT: 3, TCT: 11
Leucine	20	AAG: 4, CAG: 1, TAG: 3, CAA: 8, TAA: 4
Phenylalanine	12	GAA: 12
Asparagine	13	ATT: 4, GTT: 9
Lysine	23	CTT: 5, TTT: 18
Aspartic acid	2	GTC: 2
Glutamine	11	CTC: 5, TTC: 6
Histidine	3	GTG: 3
Glutamine	5	CTG: 2, TTG: 3
Isoleucine	38	AAT: 9, GAT: 13, TAT: 16
Methionine	14	CAT: 14
Tyrosine	22	ATA: 9, GTA: 13
Supressor	2	TTA: 2
Cysteine	8	ACA: 1, GCA: 7
Tryptophane	73	CCA: 3
Selenocysteine	6	TCA: 6

Supplementary Table 3 - Proteome Data Sources

External proteome data used in this study. Peptide sequences were either downloaded from WormBase or NCBI, in which case all sequences assigned to the given "taxid" were selected. As detailed in the main Methods section redundancy, e.g splice forms, were screened for with Cd-hit at the 99% threshold

**Species**	**Source**	**Version / Download date**	**Sequences**	**Sequences post Cd-hit %99 screen**
C. elegans	WormBase	WS233	25,848	22,855
T. spiralis	NCBI	snapshot July 2012	20,975	15,285
T. castaneum	NCBI	snapshot June 2012	26,790	19,400

Supplementary Figure 1

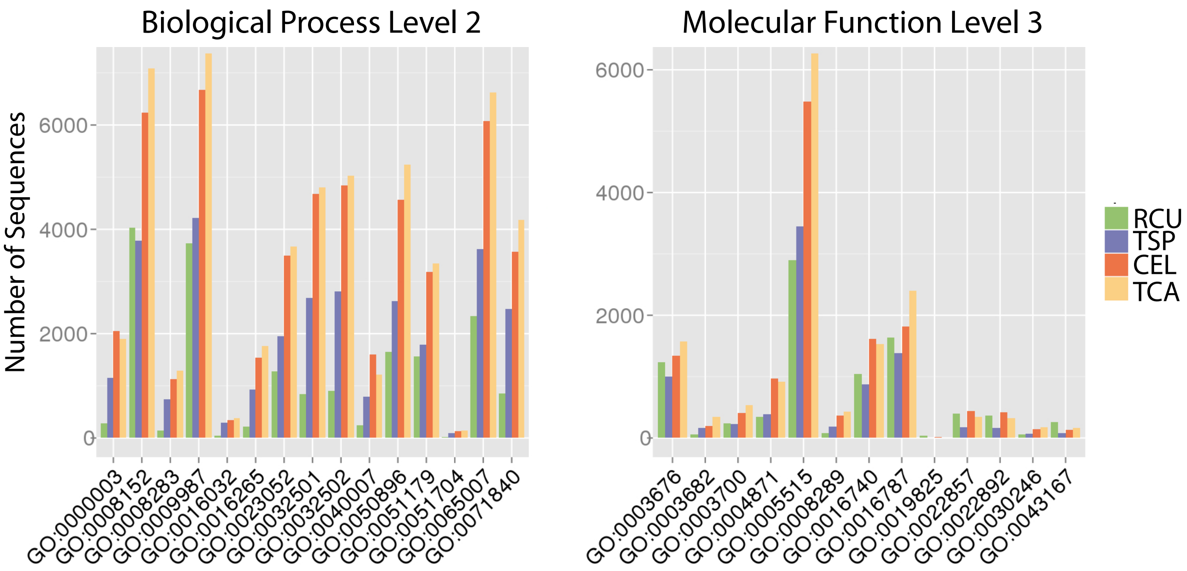

Figure SF 1: **GO slim annotation for Level 3 Molecular Function and Level 2 Biological Process.** Using the Uniprot/Swissprot database and evalue of 1e^{-5} for most reliable protein information we were able to annotate 7015 *R. culicivorax*, 5510 *T. spiralis*, 9723 *C. elegans* and 10,580 *T. castaneum*, sequences with "Biological Process" GO terms after running GO slim in Blast2GO. In the "Molecular Function" category 8465 *R. culicivorax*, 5307 *T. spiralis*, 9035 *C. elegans*and 10,414 *T. castaneum*sequences were annotated. Due to the restriction to Swissprot for most reliably curated proteins and the large sequence divergence we found between enoplean nematodes and the model organisms it is obvious that fewer proteins could be assigned with GO terms in *R. culicivorax* and *T. spiralis*, than *C. elegans* and *T. castaneum.* Hence these data, especially at informative levels of gene ontology, are rather an indication of divergence than a general key to biology go enoplean nematodes. While specific traits will have to be analysed in more detailed assays, some findings, however, can be interpreted taking the *R. culicivorax* life-cycle into account. GO:0019825 for example is of interest, as several proteins constituting the term oxygen binding narrowed to "cytochrome P450 activity" are globins in the *R. culicivorax* dataset. As described in the main text these proteins could play a role in light sensing of *R. culicivorax*, as was described for other mermithids. Additionally other functions associated with the manyfold of P450 related processes [11] could play a role in the mosquito parasite life-cycle. Such processes are for example "devoted to the metabolism of xenobiotics" [12], which could be utilised by *R. culicivorax* to defend against the host immune defense agents. Also fatty acid metabolism in which P450 plays a role [13] should be important for the adult nematodes after emergence from insect hosts when the nematodes stop feeding. Indeed upon dissection adult *R. culicivorax* specimens discharge a large number of oily droplets (own observation). As a further aspect, oxygen binding of heme containing P450 [14] could be utilized by the nematode during its under water life phases. This might as well be reflected through the GO:0043167 ("ion binding"), which appears to have more members associated in *R. culicivorax.*

Supplementary Table 4 - GO IDs and GO terms

GO IDs and corresponding GO terms for Level 3 Molecular Function and Level 2 Biological Process. See Figure SF 1.

GO-ID	GO-term: Biological Process Level 2	GO-ID	GO-term: Molecular Function Level 3
GO:0071840	cellular component organization or biogenesis	GO:0022857	transmembrane transporter activity
GO:0032501	multicellular organismal process	GO:0022892	substrate-specific transporter activity
GO:0023052	signalling	GO:0016740	transferase activity
GO:0000003	reproduction	GO:0004871	signal transducer activity
GO:0050896	response to stimulus	GO:0003700	transcription factor activity
GO:0051704	multi-organism process	GO:0005515	protein binding
GO:0008152	metabolic process	GO:0030246	carbohydrate binding
GO:0051179	localization	GO:0003682	chromatin binding
GO:0065007	biological regulation	GO:0016787	hydrolase activity
GO:0008283	cell proliferation	GO:0043167	ion binding
GO:0016032	viral reproduction	GO:0019825	oxygen binding
GO:0009987	cellular process	GO:0008289	lipid binding
GO:0016265	death	GO:0003676	nucleic acid binding
GO:0032502	developmental process		
GO:0040007	growth		

Supplementary Figure 2

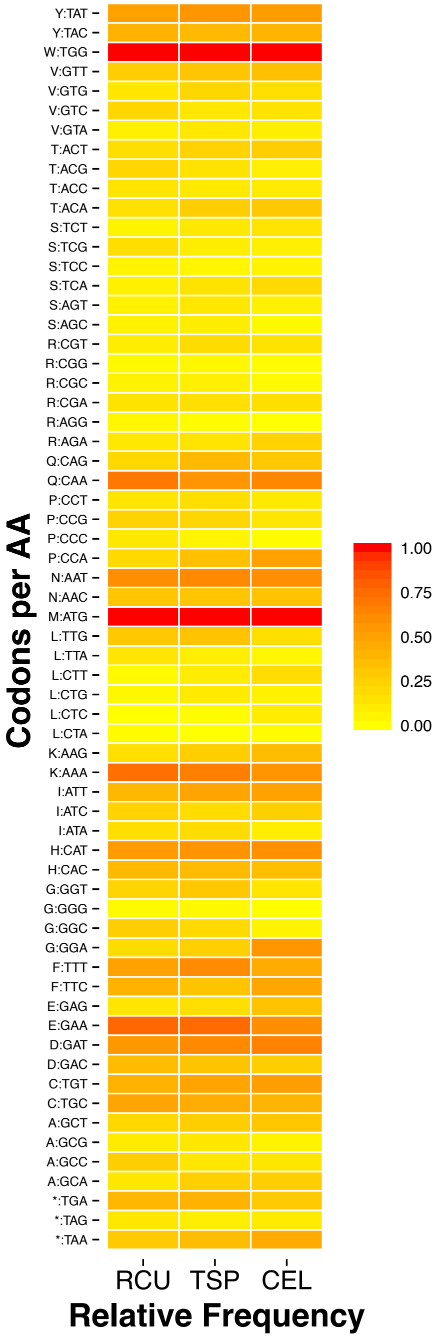

Figure SF 2: **Codon usage calculated from predicted *R. culicivorax* transcripts.** Using the full set of transcripts longer 100 codons available for each species we inferred the mean effective number of codons (ENC) and GC content at 3rd sites of fourfold degenerate amino acids employing INCA (see main text Methods). The results are in good accordance with previous results from Cutter et al. [15] for *C. elegans*. Our calculations however diverge from the published *T. spiralis* data with a mean ENC of 52.8 compared to 50.7 in the previous assay and a mean GC content at position 3 of 40% compared to 36%. This is explained due to only 2,772 *T. spiralis* genes available for analysis in [15], while we had access to the full transcriptome using 13,381 genes after filtering. Data for *C. elegans* are much more alike as the previous study could make use of 14,527 genes for the species. A mean ENC of 53.3 inferred for *R. culicivorax* is in line with the other nematodes. While the high GC content of 50% at synonymous sites found in *R. culicivorax* could indicate stronger codon bias in the species [16], it is not necessarily a consequence of selection but might be a genome wide mutation based trait [17, 18].

Supplementary References

1. Mohamed AK, Burr C, Burr AHJ: **Unique Two-Photoreceptor Scanning Eye of the Nematode *Mermis nigrescens***. *The Biological Bulletin* 2007, **212**(3):206–221.
2. Robinson AF, Baker GL, Heald CM: **Transverse Phototaxis by Juveniles of Agamermis sp. and Hexamermis sp.** *The Journal of Parasitology* 1990, **76**(2):147–152.
3. Shamseldean MM, Platzer EG: **Romanomermis culicivorax: Penetration of larval mosquitoes**. *Journal of Invertebrate Pathology* 1989, **54**(2):191–199.
4. Shamseldean MM, Platzer EG, Gaugler R: **Role of the surface coat of Romanomermis culicivorax in immune evasion**. *Nematology* 2007, **9**:17–24.
5. Burr AHJ, Wagar D, Sidhu P: **Ocellar pigmentation and phototaxis in the nematode *Mermis nigrescens*: changes during development**. *The Journal of Experimental Biology* 2000, **203**:1341–1350.
6. Burr AH, Hunt P, Wagar DR, Dewilde S, Blaxter ML, Vanfleteren JR, Moens L: **A hemoglobin with an optical function.** *The Journal of biological chemistry* 2000, **275**(7):4810–4815.
7. Hunt P, McNally J, Barris W: **Duplication and divergence: the evolution of nematode globins**. *Journal Of Nematology* 2009, **41**:35–51.
8. Schulze J, Schierenberg E: **Cellular pattern formation, establishment of polarity and segregation of colored cytoplasm in embryos of the nematode Romanomermis culicivorax.** *Developmental Biology* 2008, **315**(2):426–436.
9. Fairbank PD, Lee C, Ellis A, Hildebrand JD, Gross JM, Wallingford JB: **Shroom2 (APXL) regulates melanosome biogenesis and localization in the retinal pigment epithelium**. *Development* 2006, **133**(20):4109–4118.
10. Tang K, Xie X, Park JI, Jamrich M, Tsai S, Tsai MJ: **COUP-TFs regulate eye development by controlling factors essential for optic vesicle morphogenesis**. *Development* 2010, **137**(5):725–734.
11. Werck-Reichhart D, Feyereisen R: **Cytochromes P450: a success story**. *Genome Biology* 2000, **1**(6):3003.1–3003.9.
12. Guengerich FP: **Common and Uncommon Cytochrome P450 Reactions Related to Metabolism and Chemical Toxicity**. *Chemical Research in Toxicology* 2001, **14**(6):611–650.
13. Nelson DR: **Metazoan cytochrome P450 evolution**. *Comparative Biochemistry and Physiology Part C: Pharmacology, Toxicology and Endocrinology* 1998, **121**:15–22.
14. Groves J: **Models and mechanisms of cytochrome P450 action**. *Cytochrome P450* 2005, :1–43.
15. Cutter AD, Wasmuth JD, Blaxter ML: **The evolution of biased codon and amino acid usage in nematode genomes.** *Molecular Biology And Evolution* 2006, **23**(12):2303–2315.
16. Tiffin P, Hahn MW: **Coding Sequence Divergence Between Two Closely Related Plant Species: Arabidopsis thaliana and Brassica rapa ssp. pekinensis**. *Journal of Molecular Evolution* 2002, **54**(6):746–753.
17. Knight RD, Freeland SJ, Landweber LF: **A simple model based on mutation and selection explains trends in codon and amino-acid usage and GC composition within and across genomes**. *Genome Biology* 2001, **2**(4):research0010.1—0010.13.
18. Hershberg R, Petrov DA: **Selection on Codon Bias**. *Annual Review of Genetics* 2008, **42**:287–299.
